# Epiphora associated with anomalies of the distal end of the nasolacrimal duct in children over 12 months: endoscopic findings and treatment

**DOI:** 10.5935/0004-2749.2024-0160

**Published:** 2024-12-26

**Authors:** Patricia S. Akaishi, Antonio A. V. Cruz

**Affiliations:** 1 Department of Ophthalmology, Faculdade de Medicina de Ribeirão Preto, Universidade de São Paulo, Ribeirão Preto, SP, Brazil

**Keywords:** Lacrimal duct obstruction, Nasolacrimal duct, Silicone, Microsurgery, Endoscopy, Epiphora, Intubation, Child

## Abstract

**Purpose:**

Congenital epiphora can be related to anomalies of the nasolacrimal duct. This study aimed to assess the distal end of the nasolacrimal duct and the outcomes of endoscopic treatment in children older than 12 months with congenital epiphora.

**Methods:**

This retrospective analysis describes the clinical characteristics, management, and outcomes of symptomatic congenital lacrimal obstruction in 32 lacrimal systems of 23 children. Data was collected on the preoperative symptoms, age at the time of surgery, intraoperative findings, treatment modalities, and outcomes of the children in our cohort. All patients underwent a standard endoscopic lacrimal examination, including irrigation and diagnostic probing, viewed via the inferior meatus. Cases with complex anomalies characterized by obstructions in the canaliculi, nasolacrimal junction, or nasolacrimal duct were excluded.

**Results:**

The mean age at the time of surgery was 48.03 (±27.99) months. Four different types of distal nasolacrimal duct obstruction were diagnosed. These were obstructions by a membrane (n=12), ostium stenosis (n=15), impacted turbinate (n=3), and membranous residual flaps (n=2). They were all managed with inferior meatus microsurgery and nasal endoscopic probing without silicone intubation. After a mean follow-up period of 14.75 (±11.93) months, successful outcomes were achieved in all cases.

**Conclusion:**

Microsurgery to the inferior meatus, performed under nasal endoscopy, is a safe and effective treatment for isolated anomalies of the distal end of the nasolacrimal duct in children older than 12 months. We do not recommend silicone intubation in the absence of complex lacrimal system anomalies.

## INTRODUCTION

**Figure 1:**
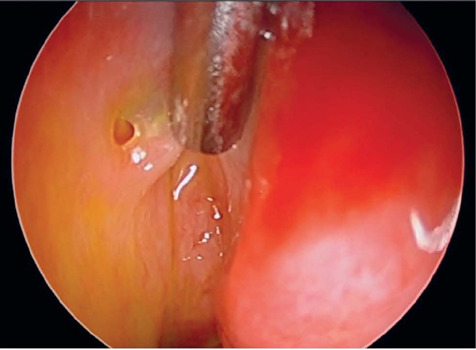
Endoscopic view of the inferior meatus and Hasner’s valve of a healthy patient. Hasner’s valve is a layer of thin tissue overlying the ostium of the nasolacrimal duct. The metal suction tube can be seen pulling the valve over the ostium.

Since the early 20^th^ century, the primary cause of epiphora in children has been nasolacrimal duct (NLD) ostium imperforation^(1)^. In most cases, canalization is completed after delivery by reabsorption or hydrostatic rupture of the distal membrane that covers the ostium^(2)^. This approach is supported by evidence from anatomical studies conducted in neonates and the high success rate of hydrostatic massage and probing^(3^,^4^,^5)^. Recent technological developments enabling direct visualization of the inferior meatus under nasal endoscopy have led to the identification of normal variations of the Hasner’s valve (Figure 1), as well as distal anomalies of the NLD related to congenital nasal duct obstruction (CNLDO). The capacity to diagnose the precise mechanism of obstruction has improved the success rate of treatment^(6^,^7^,^8^,^9^,^10^,^11^,^12)^.

In this study, we present the outcomes of our intraoperative endoscopic diagnosis and treatment protocol for distal CNLDO.

## METHODS

This investigation adhered to the tenets of the 2013 revision of the Declaration of Helsinki and was approved by the Institutional Board Review of the School of Medicine of Ribeirão Preto. The sample comprised 32 lacrimal system sides in 23 consecutive children (14 girls) diagnosed with CNLDOs between 2016 and 2019 at our hospital. The mean age of the cohort was 48.03 (±27.99) months. Diagnoses of CNLDO were made based on a medical history of epiphora and/ or discharge symptoms beginning during the neonatal period, as reported by the parents, with clinical signs of NLD obstruction, which included high tear meniscus, ocular discharge, and abnormal results on a fluorescein dye disappearance test. The diagnosis of an isolated obstruction of the distal NLD was confirmed by intraoperative examination under nasal endoscopy, according to our surgical protocol for CNLDO. Exclusion criteria were the cases diagnosed intraoperatively as combined proximal anomalies (canalicular, sac, and/ or proximal duct stenosis) were excluded. All procedures were carried out under general anesthesia by our hospital’s Oculoplastic team. Among the 23 patients, there were 2 cases of Down’s syndrome, 1 with anterior plagiocephaly, 1 with trigonocephaly, and one with Waardenburg syndrome. All patients had epiphora with (15 sides) or without (17 sides) purulent discharge, which was diagnosed by ectoscopic examination. Sac dilation was evidenced by ballooning below the medial canthus in only one side of a 12-year-old boy. Among the 32 sides, 21 (65%) had undergone failed treatments prior to surgery. These included hydrostatic massage in 16 (50%) sides, blind probing in 2 (6%) sides, endoscopic-assisted probing in 1 side (3%), and silicone intubation in 2 (6%) sides.

A successful outcome was defined as an absence of symptoms and/or no retention on the dye disappearance test (DDT) after 5 minutes.

### Statistical analysis

We used the chi-square test to determine whether there is a significant correlation between preoperative discharge and the type of distal NLD anomaly.

### Surgical technique

#### Patient preparation

Under general anesthesia, patients were prepped and draped for surgery. The inferior meatus was packed with small neurosurgical cotton pads soaked in a 1:2000 adrenaline solution for 5 minutes.

#### Intraoperative examination and surgical management protocol

**Figure 2:**
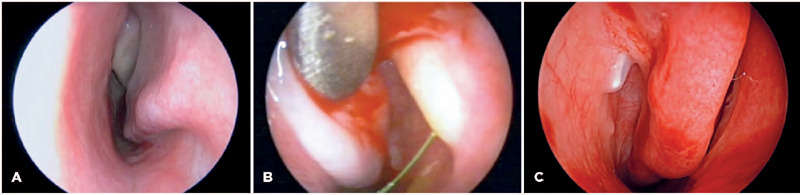
Endoscopic views of distal anomalies of the nasolacrimal duct after topical vasoconstriction and mucosa shrinkage. (A) Inferior turbinate impaction (left nostril); (B) Jet of fluorescein emerging from the stenotic ostium during irrigation. The left inferior turbinate is pushed aside by a Freer elevator; (C) A thin membrane covering the tip of a Bowman’s probe. The right inferior turbinate is subluxated (right nostril).

The inferior turbinate was examined endoscopically. When the inferior meatus was so narrow that the introduction of a Freer elevator for luxation was difficult, a diagnosis of impaction against the lateral nasal wall was made (Figure 2A). A Freer elevator was used to gently lift the turbinate toward the septum to create enough space to fully examine the inferior meatus. The examination then moved to the upper lacrimal system. Both the upper and lower puncta were dilated with a punctum dilator. The upper and lower canaliculi were then examined using a size 0 Bowman’s probe. Resistance to the free movement of the probe through the canaliculus was indicative of intracanalicular stenosis. If the probe reached the lacrimal sac and a hard stop was felt, we irrigated with diluted fluorescein in a 5-cc syringe with a lacrimal cannula. The flow into the inferior meatus was examined under nasal endoscopy. If the NLD was probed via the nasal cavity prior to lacrimal irrigation, a path to the nasal cavity was created, eliminating a diagnosis of ostium stenosis. Ostium stenosis was diagnosed when the opening was so narrow that fluorescein solution sprayed out during irrigation (Figure 2B). Depending on the diagnosis, different anomalies were managed differently. In cases of ostium stenosis, we used a Cushing nerve hook to enlarge the ostium and microforceps to remove redundant mucosa. In cases of membranous obstruction (no fluorescein seen in the meatus), the probe was pushed through the membrane (Figure 2C) which was then removed with microforceps. No silicone stent was introduced in any of the operated sides.

Postoperative care included nasal steroid spray twice daily for 1 week, saline nasal spray four times a day for 1 month, and a combination of steroidal and antibiotic eyedrops for 1 week.

## RESULTS

Four different types of distal NLD obstruction were diagnosed: membrane (n=12), ostium stenosis (n=15), impacted turbinate (n=3), and membranous residual flap (n=2). As shown in table 1, a lack of discharge was not significantly associated with ostium patency (p=0.082). In these cases, lacrimal drainage insufficiency was caused by stenosis, turbinate impaction, or a residual flap.

**Table 1:** Association between ostium patency and preoperative discharge in pediatric patients with anomalies of the distal end of the nasolacrimal duct

	Discharge +	Discharge -	Total
Patent (stenosis/impacted turbinate/flap)	7	13	20
No patent (membrane)	8	4	12

Chi-square = 3.02, p=0.082. As the significance level was p<0.05, there was no significant correlation.

After a mean follow-up of 14.75 months (±11.93), we achieved an absence of symptoms and no retention after 5 minutes on DDT in all 32 sides.

## DISCUSSION

Epiphora in children is primarily caused by imperforation of the NLD. The distal end of the lacrimal system remains occluded at birth in up to 70% of full-term newborns^(2)^. Spontaneous aperture development occurs after delivery in most cases. In the pediatric population, the prevalence of obstructive epiphora during early childhood reaches 20% in some demographics. However, the condition naturally resolves itself within the first year of life in most instances, obviating the need for surgical intervention^(13)^. Variable success rates have been reported for treatment with conventional probing in children over 12 months old, and there is insufficient evidence to endorse this clinical practice as the optimal treatment for CNLDO in older children^(14)^. Surgical treatment of CNDLO is ideally performed under general anesthesia for safety reasons, especially in children over 12 months. A complete lacrimal semiology under general anesthetic facilitates the identification of other anomalies associated with CNLDO, such as canalicular and intraductal agenesis and stenosis. Those anomalies should be treated intraoperatively when possible for optimum outcomes.

Although the internal lacrimal anatomy may be examined by dacryoendoscopy^(15^,^16^,^17^,^18)^, the NLD ostium is better visualized by nasal endoscopy.

In congenital lacrimal obstruction, nasal endoscopic examination allows clinicians to diagnostically distinguish between the distinct types of anomalies related to epiphora. In 1997, Ingels et al.^(19)^ were the first to report nasal endoscopy-guided probing in pediatric patients. Their study demonstrated the value of nasal endoscopy in the avoidance of false routes during probing. Since then, endoscopic probing has become a popular approach, and various anatomical findings have been noted in patients with NLD obstruction, including stenotic valves, membranes, and inferior turbinate impactions^(7^,^8^,^10)^. With technological advances, surgeons can now approach such anomalies directly, improving the success rates across various age groups^(11^,^20)^.

It is important to keep in mind that, although anomalies of the lacrimal ostium are the most frequent cause of congenital obstructions, malformations may be present at other sites. These can include agenesis of the canaliculus or NLD, and stenosis at the level of the common canaliculus, sac, duct, or bone. Thus, any interventions performed under general anesthesia should include a detailed evaluation of the lacrimal drainage pathway to identify and treat any other causes of obstruction intraoperatively.

Previous studies have stressed the importance of lacrimal semiology for the proper anatomical diagnosis of obstructions^(6^,^8)^. Gentle endoscopy-guided irrigation before NLD probing facilitates the diagnosis of ostium stenosis. As mentioned in the description of the surgical technique, if probing is performed at the surgical outset, a diagnosis of ostium stenosis cannot be made.

We found no significant association between discharge and obstruction type. However, our sample was small, so may have lacked sufficient statistical power.

Our study group included syndromic patients with conditions such as Down’s and Waardenburg. None of these syndromic cases had complex abnormalities of the tear duct. Cure was obtained with microsurgery of the ostium without silicone intubation in all cases. As the maxilla is not involved in any of the syndromes seen in our cohort, the syndromes cannot be independently correlated with complex tear duct abnormalities. Even in these cases, diagnostic examination is necessary to identify the most appropriate treatment.

Per our institutional protocol, we refrain from employing silicone intubation in congenital obstructions without canalicular or NLD stenosis. Silicone tubes impose additional risks, primarily in the form of postoperative complications such as tube dislodgement, punctal slitting, corneal abrasions, and granulomas^(21^,^22)^. Instances of silicone fragment rupture and retention have also been reported in the literature^(23)^. In our cohort, we found the endoscopic lacrimal approach to effectively address distal NLD obstructions. This obviated the need for silicone intubation, thereby mitigating associated complications.

Complete or incomplete distal obstruction of the NLD can be associated with epiphora and discharge in children. In most cases, an accurate diagnosis of the type of obstruction can be made intraoperatively with nasal endoscopy-guided irrigation and diagnostic probing. Using instruments designed for ear microsurgery, a ductal ostium can be created or enlarged to allow permanent free passage of tears into the nose^(24)^.

In a review of current treatments for CNLDO, Kashkouli et al. conclude that therapeutic decisions should be guided by intraoperative findings^(25)^. Based on our findings in the present study, we concur with this opinion. In adopting this approach, surgeons must be prepared to perform anything from a simple turbinate infracture to a dacryocystorhinostomy (DCR) in CNLDO patients. This avoids the need for re-treatment under anesthesia and the associated increase in cumulative costs. One-stage obstruction-based treatment guided by nasal endoscopy should be the gold standard in pediatric lacrimal surgery. Intraoperative examination facilitates the identification of the location and form of the obstruction, enabling a tailored treatment approach.

This study’s limitations included a relatively small sample size and the lack of a control group. The absence of such a group precluded the direct comparisons essential for robust analysis. Experimental endoscopic comparison of the normal anatomy of the lacrimal system in asymptomatic children with the lacrimal anatomy of children with CNLDO would be an intriguing avenue for investigation. Future studies performing such a comparison would provide valuable insights into the characteristics of the lacrimal system.

To verify our findings, further research employing our protocol, or similar protocols, should be performed with larger samples across multiple centers. This will allow definitive conclusions to be drawn regarding the goldstandard treatment for CNLDO.

Based on our results, we advocate endoscopy-guided intraoperative categorization of anatomical anomalies of the distal NLD in children older than 12 months. Using a direct endoscopic approach, surgical intervention can restore lacrimal patency to the distal end of the NLD in pediatric patients with isolated anomalies at this location.
